# Phospholipases: From Structure to Biological Function

**DOI:** 10.3390/biom11030428

**Published:** 2021-03-15

**Authors:** María A. Balboa, Jesús Balsinde

**Affiliations:** 1 Instituto de Biología y Genética Molecular, Consejo Superior de Investigaciones Científicas (CSIC), 47003 Valladolid, Spain; 2 Centro de Investigación Biomédica en Red de Diabetes y Enfermedades Metabólicas Asociadas (CIBERDEM), 28029 Madrid, Spain

Phospholipases are enzymes that cleave ester bonds within phospholipids. As a consequence of these hydrolysis reactions, a variety of lipid products are generated, which control much of cellular signaling. Phospholipase A_2_s are key enzymes in this regard due to their role as primary generators of free polyunsaturated fatty acids, which are precursors of various families of compounds playing multiple roles in inflammation [1,2,3]. Phospholipase A_2_s hydrolyze the ester bond at the sn-2 position of the glycerol backbone of phospholipids. Based on their dependence on Ca^2+^ and cellular localization, these enzymes are generally classified into several families [4,5,6]. Three of these families are the most studied in terms of cellular signaling and lipid mediator production: the Ca^2+^-dependent cytosolic phospholipase A_2_s, the Ca^2+^-dependent secreted phospholipase A_2_s, and the Ca^2+^-independent phospholipase A_2_s. In this respect, the contribution by Murakami et al. [7] provides a timely overview of the functioning of members of these three major phospholipase A_2_ families in several pathophysiological conditions, ranging from host defense and metabolism to cancer and skin barrier function.

The Ca^2+^-dependent group IVA phospholipase A_2_, also known as cytosolic phospholipase A_2_α (cPLA_2_α), is a key enzyme in inflammation because of its ability to initiate the production of eicosanoids by preferentially releasing arachidonic acid in response to cell activation. Due to this property, cPLA_2_α has been the subject of continuing pharmaceutical interest for the development of anti-inflammatory drugs. However, no phospholipase A_2_ inhibitor has yet reached the market. Targeting and inhibiting the phospholipase A_2_ reaction has proved problematic since numerous enzymes with phospholipase A_2_ activity co-exist in cells with overlapping activation properties, and the lipid metabolic pathways are complex, often redundant, and highly interconnected. Thus, the search for improved formulations continues. In this context, the work by Koutoulogenis et al. [8] shows that modifying a 2-oxoester inhibitor of cPLA_2_α by inserting a methyl group on the α-carbon atom of the oxoester results in molecules with enhanced metabolic stability that retain considerable inhibitor potency against cPLA_2_α. This increases the potential for pharmaceutical development of this class of inhibitors. In another study, Ashcroft et al. [9] show that the anti-psoriatic effects of inhibiting cPLA_2_α with the selective inhibitor AVX001, an n-3 polyunsaturated fatty acid derivative, results from a combination of anti-inflammatory and anti-proliferative effects. Thus, the therapeutic mode of action of AVX001 in psoriasis could depend both on reducing inflammatory eicosanoid production and inhibition of the hyperproliferative state of keratinocytes.

The group V secreted phospholipase A_2_ is known to participate in a number of functions of macrophages exposed to interleukin 4 (IL-4), which polarizes the cells to an anti-inflammatory phenotype. Koganesawa et al. [10] performed a mass spectrometry analysis of phospholipase A_2_ products and substrates using macrophages deficient or not in the group V enzyme, and polarized to either anti-inflammatory (IL-4-treated) or pro-inflammatory (bacterial lipopolysaccharide plus interferon-γ) phenotypes. The authors identify selective changes between the different activation regimes, suggesting the existence of novel lipid pathways and functions critical for inflammation, which may rely on the group V enzyme.

Secreted phospholipase A_2_s often act in concert with cPLA_2_α to elicit certain biological responses [11,12]. However, the molecular mechanisms involved in this cross-talk are still poorly defined. Using mass-spectrometry-based lipidomics, Rodríguez et al. [13] characterized the human monocyte response to a group IIA secreted phospholipase A_2_ from the venom of the Central American snake *Bothrops asper*. The data reveal significant interactions between the secreted phospholipase A_2_ and the cPLA_2_α of the monocytes in terms of lipid droplet biogenesis, eicosanoid responses, and biochemical pathways, which contribute to initiating the inflammatory response. Using the same secreted phospholipase A_2_ as a prototypical group IIA enzyme, Leiguez et al. [14] show that the enzyme can directly activate adipocytes to release prostaglandin E_2_ via a pathway involving cPLA_2_α activation, which in turn mediates the production of various adipokines. Thus, these findings increase our understanding of the role of group IIA secreted phospholipase A_2_ in obesity and associated metabolic disorders.

Crotoxin is a secreted phospholipase A_2_ abundantly present in the venom of the South American rattlesnake *Crotalus durissus terrificus*. Crotoxin is responsible for many of the pathological conditions associated with snakebite, including neurotoxicity, myotoxicity, and immune alterations. Sartim et al. [15] describe the effects of crotoxin on respiratory failure, and introduce the use of nicotinic blockers and prostanoid biosynthesis inhibitors as possible therapeutic agents to counteract rattlesnake envenomation.

Determining the involvement of the Ca^2+^-independent group VIA phospholipase A_2_ (commonly referred to as Ca^2+^-independent phospholipase A_2_β or iPLA_2_β) in physiology and pathophysiology has been shown to be very complex. Thanks to the generation of knock-out mice for this enzyme [16], its involvement in a variety of pathophysiological conditions could be established. In this regard, Chamulitrat et al. [17] discuss recent data with iPLA_2_β-deficient mice, implicating the enzyme in the development of hepatic steatosis and inflammation in obese and non-obese murine models. Likewise, Turk et al. [18] describe the production of mice with selective deficiency of iPLA_2_β in macrophages or insulin-secreting β cells, and the contrasting metabolic phenotype exhibited by these two kinds of cells. In studies with cultured cells, Monge et al. [19] show that in addition to cPLA_2_α, iPLA_2_β is instrumental for activated cells to mobilize adrenic acid, the two-carbon chain elongation product of arachidonic acid. As with arachidonic acid, free adrenic acid can be metabolized to a number of oxygenated bioactive metabolites. Thus, adrenic acid mobilization shares regulatory features with arachidonic acid mobilization regarding cPLA_2_α involvement, but seems to be a more complex process, as it involves participation of a second enzyme that is not involved in arachidonate release, i.e., iPLA_2_β.

Phospholipid phosphatases have recently received considerable attention because of their involvement in a number of pathophysiological states. Although not immediately recognized by some, phospholipid phosphatases are also phospholipases in their own right, as they hydrolyze the ester bond between the glycerol/sphingosine and phosphate moieties of certain phospholipids (i.e., a phospholipase C-type cleavage). Some have a very strict substrate specificity, such as the lipins, which hydrolyze phosphatidic acid only, whereas others are less specific, being able to use multiple substrates. The latter are known as lipid phosphate phosphatases, of which there are three different groups. Tang and Brindley provide a comprehensive view of what is currently known about the role of lipid phosphate phosphatases in cancer [20]. The authors discuss the structure and cellular localization of lipid phosphate phosphatases, as well as their expression levels and effects on different cancers. The authors also review the impact of two major lipid phosphate phosphatase substrates, i.e., lysophosphatidic acid and sphingosine 1-phosphate, on different aspects of tumor development.

Phosphatidic acid, the substrate of lipins, is a fundamental intermediate for the synthesis of triacylglycerol and all glycerophospholipids, and plays key roles in intracellular and intercellular signaling. The contribution of Lutkewitte and Finck [21] focuses on how the lipin-mediated control of phosphatidic acid concentrations regulates metabolism and signaling in the heart, skeletal muscle, liver, and adipose tissue. Schilke et al. [22] describe the contribution of both lipid phosphate phosphatases and lipin-1 to generate pro-inflammatory and/or pro-resolving lipid products during atherosclerosis that may influence disease progression or regression. This work is not limited to discussing the roles of phospholipid phosphatases; it also includes information on the role of other phospholipases, i.e., various members of the phospholipase A_2_ family, phospholipase C, and phospholipase D.

Majeed syndrome is a rare autoinflammatory disease that develops in patients who carry loss of function mutations in the *LPIN2* gene, which encodes for lipin-2. Ferguson and El-Shanti [23] present a comprehensive and up-to-date review of this disorder, emphasizing clinical features, genetics, pathogenesis, and cellular and molecular mechanisms. The latter include exacerbated activation of the NLPR3 inflamasome in macrophages, as well as increased production of osteoclastogenic mediators by M2-polarized macrophages.

Sphingomyelinases are a special group of phospholipase C-type enzymes that specifically hydrolyze sphingomyelin to generate ceramide, a potent inductor of apoptosis. Cells can evade apoptosis by converting ceramide to sphingosine-1-phosphate. In this regard, Hawkins et al. [24] discuss the role of sphingomyelinases and sphingosine 1-phosphate in glioblastoma and metastatic brain tumors. There are two main types of sphingomyelinases: neutral and acid. Both have been well-characterized for their contribution to signaling pathways and their roles in diverse pathologies, including liver diseases. Insausti-Urkia et al. [25] summarize the physiological functions of neutral and acid sphingomyelinases and their role in chronic and metabolic liver diseases.

Collectively, the papers included in this Special Issue provide forefront information related to the role of phospholipases in a number of physiological and pathophysiological settings. It is expected that these novel and exciting findings will help stimulate further interest in the highly competitive and rapidly moving field of lipid signaling.
